# Impact of Treatment Delay on the Prognosis of Patients with Ovarian Cancer: A Population-based Study Using the Surveillance, Epidemiology, and End Results Database

**DOI:** 10.7150/jca.87881

**Published:** 2024-01-01

**Authors:** Jing Zhao, Ruiying Chen, Yanli Zhang, Yu Wang, Haiyan Zhu

**Affiliations:** Department of Shanghai Key Laboratory of Maternal Fetal Medicine, Shanghai Institute of Maternal-Fetal Medicine and Gynecologic Oncology, Shanghai First Maternity and Infant Hospital, School of Medicine, Tongji University, Shanghai, China.

**Keywords:** Ovarian cancer, Treatment delay intervals, SEER, Prognosis, Retrospective

## Abstract

**Purpose:** This study aimed to assess the impact of treatment delay on prognosis in patients with ovarian cancer.

**Methods:** A retrospective analysis of patients with ovarian cancer in the Surveillance, Epidemiology, and End Results (SEER) database between 2010 and 2015 was performed. Chi-square tests were used to assess baseline differences. The Kaplan-Meier method was used to evaluate the effect of different treatment intervals on survival outcomes in patients. Cox regression analyses were used to identify independent factors associated with ovarian cancer prognosis.

**Results:** Of the 21,590 patients included, 15,675 (72.6%), 5,582 (25.9%), and 333 (1.54%) were classified into the immediate-treatment (<1 month after diagnosis), intermediate-delay (1-2 month delayed), and long-delay groups (≥3 months delayed), respectively. The 5-year probability of overall survival (OS) was 61.4% in the immediate-treatment group, decreasing to 36.4% and 34.8% in the intermediate- and long-delay groups, respectively. Similar survival differences were also reflected in cancer-specific survival (CSS), with 5-year CSS probabilities of 66.7%, 42.6%, and 41.8% in the aforementioned groups, respectively. Patients in the intermediate-delay group showed poorer OS (adjusted hazard ratio [HR], 1.06; 95% confidence interval [CI], 1.02-1.11; p=0.006) and CSS (adjusted HR, 1.06; 95% CI, 1.01-1.11; p=0.012) than immediate-treatment group.

**Conclusions:** Patients with delayed treatment had poorer OS and CSS. The patient's waiting time from diagnosis to initial treatment should be within 1 month.

## Introduction

According to the latest statistics, there were approximately 12,810 deaths and 19,880 new diagnoses of ovarian cancer in the United States in 2022 1. Despite improvements in therapeutics, the 5-year overall survival (OS) rate remains below 50% 2. Several studies have reported a number of clinical features that may influence the prognosis of ovarian cancer 3-6. Delays in cancer treatment are common and were particularly common during the COVID-19 pandemic. Whether delaying treatment affects the clinical outcome of ovarian cancer is currently unclear. A study looking at different referral methods for patients with ovarian, lung, prostate, and colorectal cancer found that delayed referral had no effect on survival for ovarian cancer patients 7. Pyeon et al. 8 reported that delaying palliative chemotherapy had no adverse effects on the survival of patients with recurrent ovarian cancer. Delayed chemotherapy had no effect on the chemotherapy efficacy and declined levels of cancer antigen (CA) 125 during the coronavirus disease 2019 (COVID-19) pandemic 9.

Clearly, few studies have reported the impact of delayed treatment on the prognosis of patients with ovarian cancer, and few criteria are currently available for assessing the time intervals associated with delayed treatment in patients with ovarian cancer. Using the national sample of patient data obtained from the Surveillance, Epidemiology, and End Results (SEER) database, this study analyzed the detailed information of patients with delayed treatment. The aim is to assess the detrimental effect of delayed treatment on patient outcomes, as well as the patient characteristics for which delayed treatment has a greater impact on prognosis, in order to provide new guidance for treatment.

## Methods

### Data and Variables

**Figure 1:**
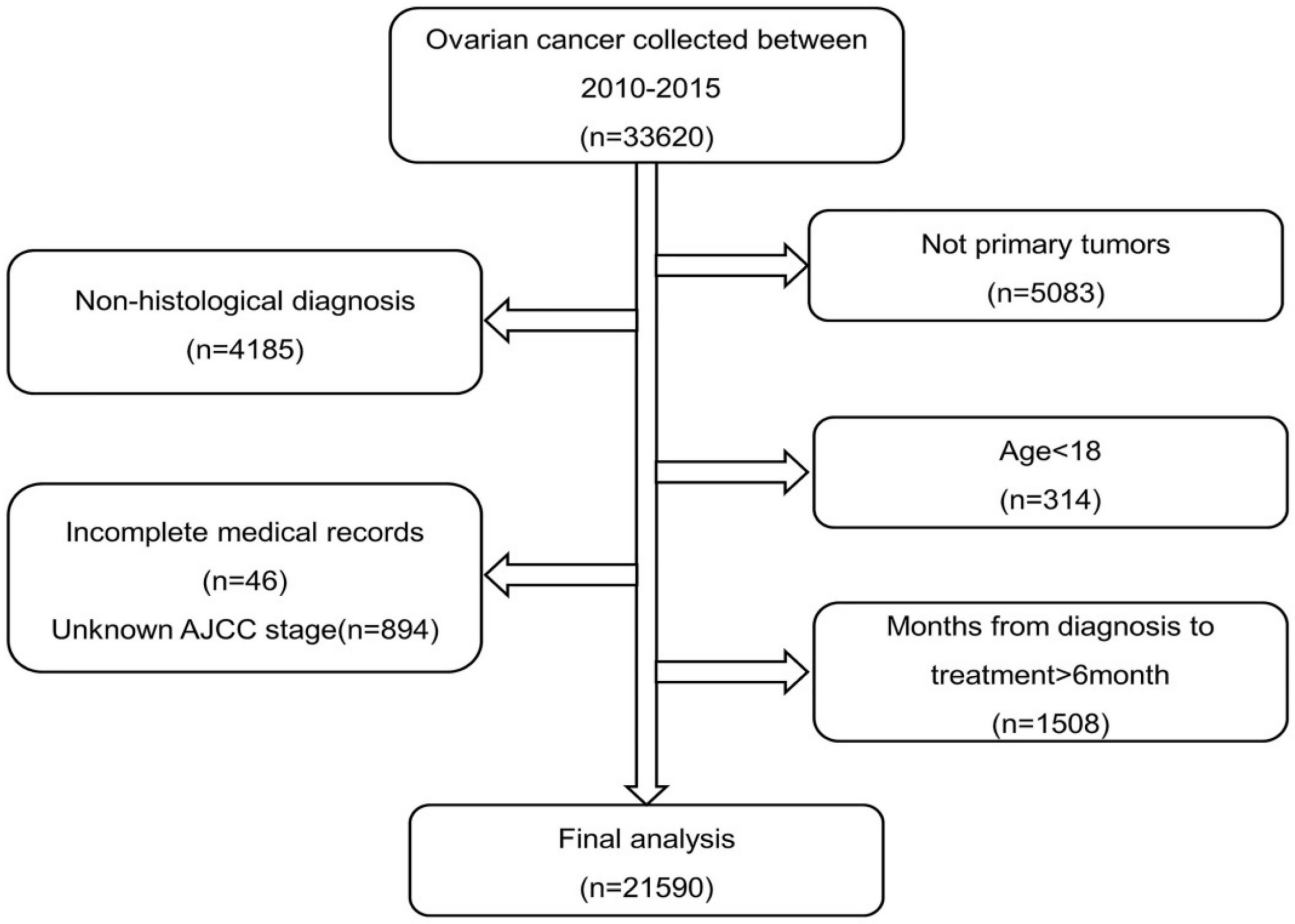
Flow chart of patient selection from the SEER database

SEER*Stat version 8.4.0.1 (https://seer.cancer.gov/seerstat/) was used to obtain patient data from SEER 17 Registries Database during 2010 to 2015. The inclusion criteria were as follows: (1) patients with ovarian cancer initially screened using the International Classification of Oncological Diseases 3 (ICD-O-3) code whose diagnosis was confirmed pathologically and (2) patients with primary ovarian cancer as their first cancer. The exclusion criteria were as follows: (1) patients survival time was 0, (2) patients with incomplete histological and staging information, (3) patients aged <18 years, and (4) treatment delays of >6 months owing to statistically invalid results because of the small sample size. From the SEER database, we initially extracted 28,537 patients diagnosed with ovarian cancer. A total of 6,947 patients were excluded; hence, 21,590 patients were finally included. The detailed exclusion criteria is shown in Figure 1. The tumor stage of patients was extracted according to the American Joint Committee on Cancer (AJCC), 7^th^ edition. The ICD-O-3 code was selected to identify histological types.

Variables, including age, marital status, race, rural/urban, median household income, regional lymph node examination, distant metastasis, stage, histological type, grade of differentiation, therapy, residual tumor and surgery were included in the study. Race was classified as white, black, or other/unknown (e.g., Hispanic, Asian, and so on). The category 'other epithelial disorders' contains less specific and/or diagnostic combinations. The therapy was classified as 'neoadjuvant chemotherapy (NACT)', 'primary surgical therapy', or 'no chemotherapy and/or surgery'. The surgery was classified as 'local resection', 'debulking surgery', 'pelvic exenteration', or 'Unknown'. The residual tumor was classified as 'no residual lesion', '≤1 cm', '>1 cm', or 'Unknown'.

The data on treatment time intervals in this study were from the variable 'Months from diagnosis to treatment' in the SEER database. The database handbook states that treatment could include surgery, radiation therapy, chemotherapy, hormone, immunotherapy, and/or active surveillance. Currently, there are no guidelines or consensus defining a specific time interval for treatment delays. According to previous studies 10-12, an initial treatment of ≥1 month is considered delayed treatment, whereas an interval of ≥3 months is considered a long-delay treatment. We divided patients into three groups: immediate-treatment (<1 month), intermediate-delayed (1-2 months), and long-delay (≥3 months).

### Statistical analysis

The Chi-square test and Kruskal-Wallis test were used to detect variables among the three groups. Univariate and multivariate Cox regression analyses were used to identify independent risk factors. Kaplan-Meier analyses were used to calculate survival rates. SPSS version 22.0 (IBM Corp., Armonk, NY, USA) and R version 4.2.2 (www.r-project.org) were used for statistical analyses. A two-tailed p-value <0.05 was considered statistically significant.

## Results

### Demographic and clinical characteristics

The baseline data of 21,590 patients are compared in Table 1. Of these patients, 15,675 (72.6%), 5582 (22.7%), and 333 (1.5%) received immediate-treatment (<1 month), intermediate-delay treatment (1-2 months) and long-delay treatment (≥3 months) following diagnosis. Age, marital status, race, regional lymph node examination, distant organ metastasis, stage, histology, grade, surgery, therapy and residual status significantly differed across the three groups. The median age of patients in the immediate-treatment, intermediate-delay treatment, and long-delay treatment groups was 58 (interquartile range [IQR], 49-67), 64 (IQR, 55-72), and 63 (IQR, 54-71) years (p<0.001), respectively. In the long-delay group, single patients comprised 57.7%, whereas married patients accounted for only 39.0% (p<0.001). The distribution of different races was different among the three groups (p <0.001). Differences were also observed during lymph node examination: 43.1% of patients in the immediate-treatment group had negative regional lymph node examination results (p <0.001). Moreover, we noted differences in the treatment times on distant metastases. In the immediate-treatment group, patients without distant metastasis comprised 83.6% of the cohort, whereas patients with distant metastasis accounted for only 15.6%. In the intermediate- and long-delayed groups, the number of patients without metastasis dropped to 58.2% and 54.4% (p <0.001), respectively. Most patients with advanced stages experienced delayed treatment. Among patients with intermediate-delay treatment, stage III and IV patients comprised 44.8% and 40.9% of the cohort, respectively; stage III and IV patients comprised 37.8% and 42.9% of those who underwent long-delay treatment (p <0.001), respectively. Histology was differentially distributed among the three cohorts (p <0.001). Approximately 50% of patients (range, 44.4-53.1%) had poorly differentiated/undifferentiated tumor status, and tissue differentiation was significantly different among the three groups (p <0.001). Of 38.1% patients in the immediate-treatment group received debulking surgery, while 50.1% of patients in the intermediate-delayed treatment group received debulking surgery (P <0.001). More than half (62.6%) of the patients in the immediate-treatment group received primary surgical therapy, and only 8.5% of the patients received NACT (P <0.001). There was a difference in residual tissue size between the three groups of patients (p < 0.001).

Overall, in the immediate-treatment group, the predominant ethnic group was White (67.3%), and the immediate-treatment group had the youngest patient (age, 58 [IQR, 49-67]), highest survival rate (56.1%), and lowest death rate from ovarian cancer (35.8%).

### Effect of treatment delay on OS

**Figure 2:**
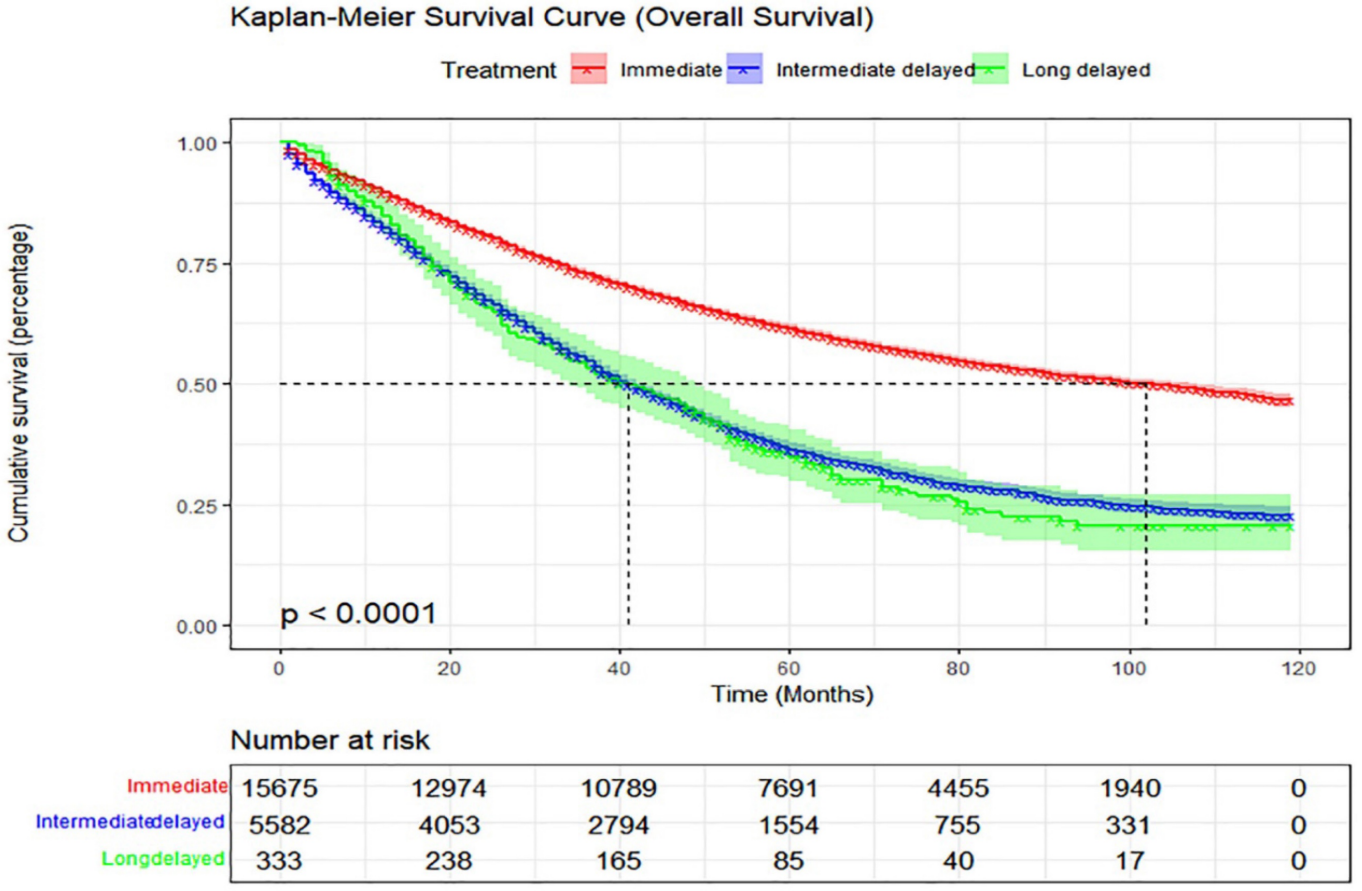
Kaplan-Meier curve of overall survival according to months from diagnosis to treatment

The study population from the SEER database was followed up until November 2021. A total of 10,922 (50.6%) patients died, and the median survival time was 54 (IQR, 25-79) months. The 2-year survival rate of all patients was 87.0%, the 5-year survival rate was 54.5%, and the 10-year survival rate was 40.0%. The median survival times in the immediate, intermediate-delay, and long-delay treatment groups were 59, 40, and 39 months, respectively. The 5-year OS rates of patients in the aforementioned groups were 61.4%, 36.4%, and 34.8%, respectively (p <0.001) (Figure 2).

Subsequently, we explored the factors affecting OS in ovarian cancer using univariate and multivariate Cox regression. Months from diagnosis to treatment, age, race, marital status, household income, tissue differentiation, stage, histology, therapy and residual tumor were independent risk factors of OS in patients with ovarian cancer (Table 2). Of note, after adjusting for other confounders, the intermediate-delay group (1-2-month delay) showed a worse OS compared with the immediate-treatment group (adjusted hazard ratio [HR], 1.06; 95% confidence interval [CI], 1.02-1.11; p=0.005) (Table 2).

### Effect of treatment delay on cancer-specific survival (CSS)

**Figure 3:**
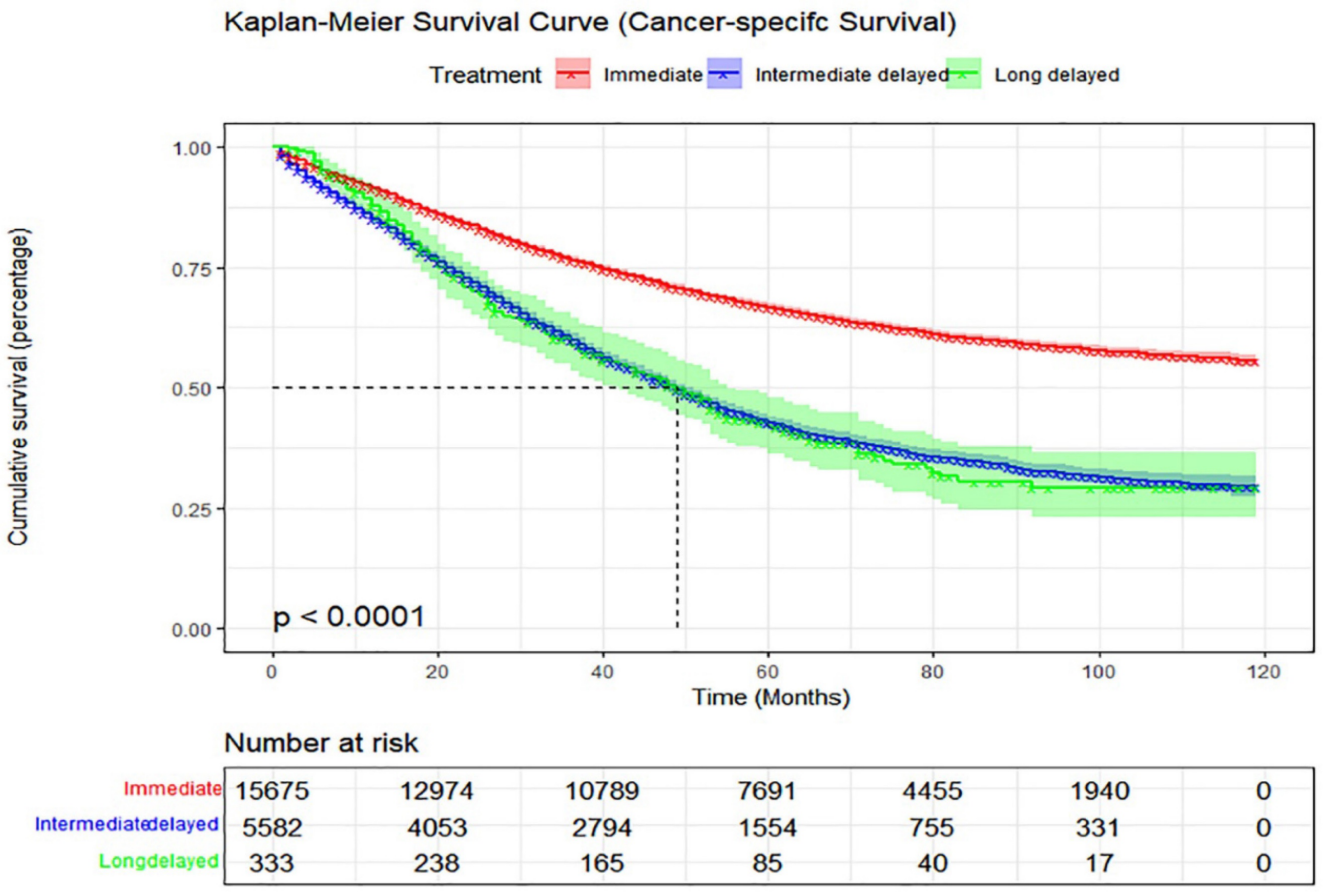
Kaplan-Meier curve of cancer-specific survival according to months from diagnosis to treatment

The 5-year CSS rates also differed significantly among the three groups, with rates of 66.7%, 42.6%, and 41.8%, respectively (Figure 3). We explored the factors affecting CSS in patients with ovarian cancer using univariate and multivariate Cox analyses. Months from diagnosis to treatment, age, race, marital status, household income, tissue differentiation, stage, histology, therapy and residual were identified as independent predictors of CSS (Table 3). After adjusting for interference factors, the intermediate-delay (1-2-month delay) group had significantly impaired CSS (adjusted HR, 1.06; 95% CI, 1.01-1.11; p=0.010) (Table 3).

### Survival at different treatment delay intervals after subgroup stratification

We performed a stratified analysis based on histological type and stage (Table 4). Compared with the immediate-treatment group, among patients with serous tumor stage I/II, the long-delay group had a 2.65 times higher risk of mortality (OS: HR, 2.65; 95% CI, 1.40-5.01; p =0.003) and a 2.41 times higher risk of cancer-specific mortality (CSS: HR, 2.41; 95% CI, 1.13-5.17; p=0.023), and among patients with serous ovarian tumor stage III/ IV, both the mortality and cancer-specific mortality risk rate in the intermediate-delay group increased (OS: HR, 1.09; 95% CI, 1.04-1.15; p<0.001; CSS:HR, 1.09; 95% CI, 1.03-1.15; p=0.002) (Table 4). The same was true for patients with clear cell tumor stage I/II (Table 4).

**Figure 4:**
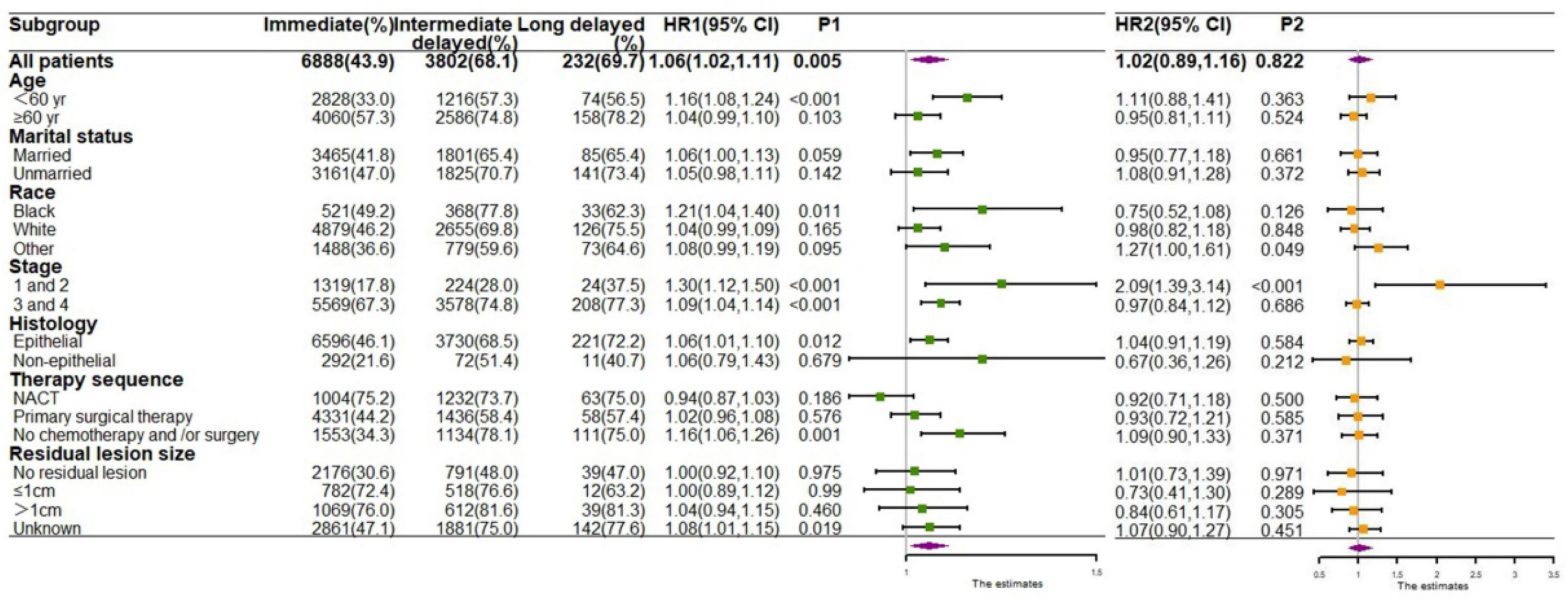
Forest plots for subgroup analysis of OS. HR1 P1 HR of the intermediate-delay group compared with that of the immediate treatment group. HR2 P2 HR of the long-delay group compared with that of the immediate treatment group.

**Figure 5:**
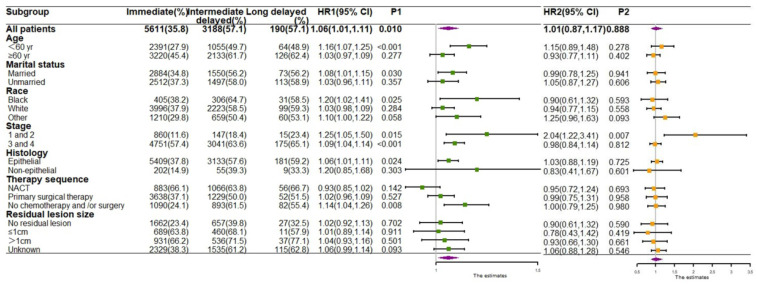
Forest plots for subgroup analysis of CSS. HR1 P1 HR of the intermediate-delay group compared with that of the immediate treatment group. HR2 P2 HR of the long-delay group compared with that of the immediate treatment group.

Moreover, to describe which characteristics of patients with delayed treatment lead to a worse prognosis, we performed stratified analyses based on both demographic and clinical characteristics to observe the OS (Figure 4) and CSS (Figure 5) of patients. For patients aged <60 years, who were black, were in stage I/II, had epithelial ovarian tumors, and had not undergo chemotherapy and/or surgery, both OS and CSS risks were significantly increased if they received intermediate- delay treatment. Additionally, the highest mortality and cancer-specific mortality risk values were observed in the long-delay group in patient with stage I/II (OS: HR, 2.09; 95% CI, 1.39-3.14; p <0.001; CSS:HR, 2.04; 95% CI, 1.22-3.41; p=0.007) (Figure 4 and Figure 5).

## Discussion

Delays in cancer treatments are common, and treatment may be delayed for reasons such as insurance, seeking a second opinion, imaging evaluation, referral and prehabilitation. A retrospective study of 1,463 ovarian cancer patients by Nagle et al. 13 found that once symptoms of ovarian cancer appear, delay in diagnosis does not adversely affect survival. A systematic review covering various types of cancer comprehensively analyzed 209 trials in 177 articles and concluded that timely diagnosis and treatment of symptomatic cancer patients can improve the survival rate of patients 14. Noer MC et al. 15 reported that the impact of ovarian cancer comorbidities on survival appears to be independent of systemic delays. More recently, Sud, A et al. 16 reported that a delay of 3/6 months in surgery for incident cancers would result in a 19%/43% reduction in life years gained. Overall, there is currently no consensus on the relationship between the timing of ovarian cancer patients' initial treatment and their prognosis. Importantly, due to reduced travel and controlled administration, delays in treatment were more pronounced during the COVID-19 pandemic 17. Therefore, insight into the prognostic impact of treatment delays is essential.

Our study assessed the impact of treatment interval on the prognosis of ovarian cancer using data from a national population-based database; we found that delayed treatment was an independent risk factor for ovarian cancer prognosis. The 5-year probability of OS was 61.4% in the immediate-treatment group, while it decreased sharply to 36.4% and 34.8% in the intermediate- and long-delay groups, respectively. Similarly, the 5-year CSS also showed a downward trend when switching between the three groups, with rates of 66.7%, 42.6% and 41.8%, respectively. In the multifactorial Cox analysis, the mortality risks of OS and CSS were higher in the intermediate-delay group than in the immediate-treatment group. Similarly, elevated risk values for OS and CSS were observed in the long-delay group; however, no statistically significant results could be calculated owing to the small sample size of this cohort. In summary, our study shows that patients with ovarian cancer are at an increased risk of mortality if their treatment is delayed for over 1 month since diagnosis. Several studies have reported the adverse effects of delayed treatment on the prognosis of patients with breast, liver, and colorectal tumors, and our results are consistent with these findings 11, 18-21. An analysis by Hanna et al. 22 of seven types of cancers revealed that the mortality risk increases by 6-8% for every month surgery is postponed. Minami et al. 23 found that delayed surgical treatment slightly affected the pathological staging of patients with ductal carcinoma in situ but did not affect OS. Another study suggests that delay has the least effect on sub-centimeter nodules and may have the greatest effect on stage II disease 24. We speculate that such an effect of delay is related to clinical characteristics and disease stage.

There is a correlation between age and treatment delay, and similar observations have been made in other studies, showing that treatment refusal rates increase with age among patients with cancer 25. There are also racial and ethnic differences in treatment delays 20. Black patients have a higher probability of delaying initial surgery and chemotherapy treatment 26-28, which may be associated with specific social factors and, ultimately, lead to an increased risk of death 29, 30. Marital status has also been associated with treatment delay, with married patients having shorter waiting intervals from diagnosis to treatment 10. This may be related to the psychosocial support and economic support of their spouse, leading to more aggressive visits to the hospital to actively seek help and cooperate to undergo early treatment.

The histology and stage are important factors affecting the prognosis of patients with ovarian cancer; therefore, we performed a stratified analysis based on these two factors. We found that delays in treatment significantly impaired the prognosis of patients with serous tumors and stage I/II clear cell tumors. In further stratified analysis according both demographic and clinical characteristics, we found that patients aged <60 years, who are black, are in stage I/II, with epithelial tumors and not receiving chemotherapy and/or surgery are more clinically beneficial if treated immediately. If these patients underwent intermediate-delay treatment, the risk of death would be increased relative to other subgroups of patients. The results suggest the importance of early and aggressive intervention as clinicians can standardize the time window for patients who must wait for treatment based on these results and manage these patients more promptly. In addition, prehabilitation can improves tolerance during surgery and chemotherapy 10, women with advanced disease, advanced age, and comorbidities who are unable to tolerate major upfront surgery may be able to delay surgery by undergoing prehabilitation prior to surgery.

Ovarian cancer has the lowest 5-year survival rate among all gynecological tumors. The current mainstream treatment paradigm is dedicated to the development of novel therapeutic approaches. The implications of the results of our study are that, if the time window for the initial treatment of ovarian cancer is reduced to within 1 month, survival optimization may be achieved. This evidence also has important socioeconomic value, as gains in improved survival from shortening the time between diagnosis and initiation of treatment may be comparable to or greater than the benefits of developing some of the novel therapeutic agents 31, 32.

The present study had some limitations. First, there were potential confounding factors and selective bias in the data processing. Second, we currently have limited information on delayed treatment. Limitations of the SEER database prevented us from collecting specific patterns of initial treatment related to the time interval from diagnosis to treatment. Third, the long-term delayed treatment group had fewer patients and low statistical testing power, so the generalisation of the conclusions remains cautious. Finally, the SEER database is based on data from US registries, and it is unclear whether the results are applicable to other countries or regions.

## Conclusions

Our study shows that prolonged initial treatment time is associated with poor prognosis in ovarian cancer patients. We recommend that ovarian cancer be treated within 1 month of diagnosis. Still, causality should be taken with caution, given the source of the data and the vast factors affecting treatment delays and prognosis in ovarian cancer. Gynecological oncologists should be aware of the impact of delayed treatment on the subsequent survival of patients with ovarian cancer and ensure timely treatment of patients after diagnosis to improve patient survival outcomes.

## Figures and Tables

**Table 1:** Baseline characteristics of patients with different time delay intervals 2010 to 2015(n=21590)

Variables	Immediate n=15675	Intermediate -delay n=5582	Long-delay n=333	P-value
Age	58(IQR, 49-67)	64(IQR, 55-72)	63(IQR,54-71)	<0.001
Marriage				<0.001
Married	8288(52.9)	2756(49.4)	130(39.0)	
Single	6730(42.9)	2582(46.3)	192(57.7)	
Unknown	657(4.2)	244(4.4)	11(3.3)	
Race				<0.001
Black	1059(6.8)	473(8.5)	53(15.9)	
White	10554(67.3)	3802(68.1)	167(50.2)	
Other	4062(25.9)	1307(23.4)	113(33.9)	
Rural-Urban				0.091
Metropolitan	14091(89.9)	4996(89.5)	310(93.1)	
Nonmetropolitan	1573(10.0)	577(10.3)	23(6.9)	
Unknown	11(0.1)	9(0.2)	0(0.0)	
Median household income inflation				0.091
< $35,000	227(1.4)	70(1.3)	4(1.2)	
$35,000 - $55,000	3001(19.1)	1069(19.2)	68(20.4)	
$55,000 - $75,000	7623(48.6)	2786(49.9)	181(54.4)	
>$75,000	4824(30.8)	1657(29.7)	80(24.0)	
Regional nodes				<0.001
Negative	6761(43.1)	1270(22.8)	75(22.5)	
Positive	2590(16.5)	1054(18.9)	58(17.4)	
Not detected	6207(39.6)	3190(57.1)	196(58.9)	
Unknown	117(0.7)	68(1.2)	4(1.2)	
Metastases at distance				<0.001
Negative	13103(83.6)	3249(58.2)	181(54.4)	
Positive	2447(15.6)	2284(40.9)	143(42.9)	
Unknown	125(0.8)	49(0.9)	9(2.7)	
Stage				<0.001
I	5600(35.7)	486(8.7)	42(12.6)	
II	1797(11.5)	314(5.6)	22(6.6)	
III	5831(37.2)	2498(44.8)	126(37.8)	
IV	2447(15.6)	2284(40.9)	143(42.9)	
Histology				<0.001
Serous	9000(57.4)	4365(78.2)	229(68.8)	
Mucinous	1134(7.2)	160(2.9)	18(5.4)	
Endometrioid	1983(12.7)	298(5.3)	21(6.3)	
Clear	1272(8.1)	206(3.7)	14(4.2)	
Other epithelial	931(5.9)	413(7.4)	24(7.2)	
Non-epithelial	1355(8.6)	140(2.5)	27(8.1)	
Grade				<0.001
Well differentiated	1589(10.1)	206(3.7)	17(5.1)	
Moderately differentiated	2179(13.9)	406(7.3)	26(7.8)	
Poorly differentiated/undifferentiated	8326(53.1)	3233(57.9)	148(44.4)	
Unknown	3581(22.8)	1737(31.1)	142(42.6)	
Cause of death				<0.001
Alive	8787(56.1)	1780(31.9)	101(30.3)	
Ovarian cancer	5611(35.8)	3188(57.1)	190(57.1)	
Other cause	1277(8.1)	614(11.0)	42(12.6)	
Survival months	59(IQR,30-83)	40(IQR,18-63)	39(IQR,18-60.5)	<0.001
Surgery				<0.001
Local resection	8789(56.1)	1695(30.4)	126(37.8)	
Debulking surgery	5967(38.1)	2795(50.1)	110(33.0)	
Pelvic exenteration	243(1.6)	99(1.8)	6(1.8)	
Unknown	676(4.3)	993(17.8)	91(27.3)	
Therapy				<0.001
NACT	1336(8.5)	1671(29.9)	84(25.2)	
Primary surgical therapy	9809(62.6)	2458(44.0)	101(30.3)	
No chemotherapy and/or surgery	4530(28.9)	1453(26.0)	148(44.4)	
Residual				<0.001
No residual lesion	7105(45.3)	1649(29.5)	83(24.9)	
≤1cm	1080(6.9)	676(12.1)	19(5.7)	
>1cm	1407(9.0)	750(13.4)	48(14.4)	
Unknown	6083(38.8)	2507(44.9)	183(55.0)	

**Abbreviation** NACT: Neoadjuvant chemotherapy

**Table 2:** Univariate and multivariate analyses of the association of treatment delay intervals with overall survival (OS)

	Univariate analysis		Multivariate analysis	
Variables	HR (95% CI)	P-value	HR (95% CI)	P-value
Months from diagnosis to treatment				
Immediate	1.00(ref)	ref	1.00(ref)	ref
Intermediate-delay	1.99(1.91,2.07)	<0.001	1.06(1.02,1.11)	0.005
Long-delay	2.08(1.82,2.37)	<0.001	1.02(0.89,1.16)	0.822
Age	1.04(1.04,1.04)	<0.001	1.02(1.02,1.02)	<0.001
Race				
Black	1.00(ref)	ref	1.00(ref)	ref
White	0.81(0.76,0.87)	<0.001	0.82(0.77,0.88)	<0.001
Other	0.65(0.60,0.70)	<0.001	0.81(0.75,0.88)	<0.001
Rural-Urban				
Metropolitan	1.00(ref)	ref	1.00(ref)	ref
Nonmetropolitan	1.14(1.07,1.21)	<0.001	1.01(0.95,1.09)	0.688
Unknown	1.52(0.90,2.57)	0.114	1.28(0.76,2.17)	0.360
Marriage				
Married	1.00(ref)	ref	1.00(ref)	ref
Single	1.22(1.17,1.26)	<0.001	1.16(1.11,1.20)	<0.001
Unknown	1.06(0.96,1.17)	0.227	0.98(0.89,1.08)	0.750
Median household income inflation				
< $35,000	1.00(ref)	ref	1.00(ref)	ref
$35,000 - $55,000	0.90(0.77,1.05)	0.177	0.96(0.82,1.13)	0.623
$55,000 - $75,000	0.78(0.67,0.91)	0.001	0.86(0.73,1.02)	0.079
>$75,000	0.73(0.62,0.85)	<0.001	0.83(0.70,0.98)	0.026
Stage				
I	1.00(ref)	ref	1.00(ref)	ref
II	2.80(2.54,3.10)	<0.001	2.80(2.53,3.11)	<0.001
III	6.91(6.43,7.43)	<0.001	5.93(5.45,6.45)	<0.001
IV	11.34(10.52,12.22)	<0.001	8.23(7.53,8.99)	<0.001
Histology				
Serous	1.00(ref)	ref	1.00(ref)	ref
Mucinous	0.34(0.31,0.38)	<0.001	1.49(1.32,1.68)	<0.001
Endometrioid	0.27(0.25,0.30)	<0.001	0.84(0.76,0.92)	<0.001
Clear	0.49(0.45,0.54)	<0.001	1.39(1.27,1.52)	<0.001
Other epithelial	1.42(1.33,1.52)	<0.001	1.44(1.35,1.55)	<0.001
Non-epithelial	0.32(0.29,0.36)	<0.001	0.95(0.85,1.06)	0.363
Grade				
Well differentiated	1.00(ref)	ref	1.00(ref)	ref
Moderately differentiated	2.23(1.95,2.55)	<0.001	1.66(1.44,1.90)	<0.001
Poorly differentiated/undifferentiated	5.16(4.58,5.82)	<0.001	1.98(1.74,2.25)	<0.001
Unknown	4.90(4.33,5.54)	<0.001	1.88(1.65,2.14)	<0.001
Therapy				
NACT	1.00(ref)	ref	1.00(ref)	ref
Primary surgical therapy	0.50(0.47,0.52)	<0.001	0.91(0.86,0.96)	<0.001
No chemotherapy and/or surgery	0.54(0.51,0.57)	<0.001	1.50(1.41,1.59)	<0.001
Residual				
No residual lesion	1.00(ref)	ref	1.00(ref)	ref
≤1cm	3.00(2.81,3.20)	<0.001	1.57(1.47,1.68)	<0.001
>1cm	3.48(3.28,3.70)	<0.001	1.74(1.63,1.85)	<0.001
Unknown	2.11(2.02,2.21)	<0.001	1.45(1.38,1.52)	<0.001

**Abbreviation** NACT: Neoadjuvant chemotherapy

**Table 3:** Univariate and multivariate analyses of the association of treatment delay intervals with cancer-specific survival (CSS)

	Univariate analysis		Multivariate analysis	
Variables	HR (95% CI)	P-value	HR (95% CI)	P-value
Months from diagnosis to treatment				
Immediate	1.00(ref)	ref	1.00(ref)	ref
Intermediate-delay	2.03(1.95,2.12)	<0.001	1.06(1.01,1.11)	0.010
Long-delay	2.07(1.79,2.40)	<0.001	1.01(0.87,1.17)	0.888
Age	1.03(1.03,1.04)	<0.001	1.02(1.01,1.02)	<0.001
Race				
Black	1.00(ref)	ref	1.00(ref)	ref
White	0.83(0.77,0.90)	<0.001	0.84(0.78,0.91)	<0.001
Other	0.66(0.61,0.72)	<0.001	0.82(0.76,0.90)	<0.001
Rural-Urban				
Metropolitan	1.00(ref)	ref	1.00(ref)	ref
Nonmetropolitan	1.12(1.05,1.20)	0.001	1.02(0.94,1.10)	0.679
Unknown	1.31(0.71,2.44)	0.389	1.09(0.59,2.04)	0.779
Marriage				
Married	1.00(ref)	ref	1.00(ref)	ref
Single	1.16(1.11,1.21)	<0.001	1.13(1.08,1.18)	<0.001
Unknown	1.02(0.92,1.14)	0.702	0.96(0.86,1.07)	0.485
Median household income inflation				
< $35,000	1.00(ref)	ref	1.00(ref)	ref
$35,000 - $55,000	0.90(0.76,1.06)	0.212	0.95(0.79,1.13)	0.541
$55,000 - $75,000	0.80(0.68,0.95)	0.010	0.88(0.73,1.05)	0.160
>$75,000	0.74(0.62,0.87)	<0.001	0.83(0.69,0.99)	0.042
Stage				
I	1.00(ref)	ref	1.00(ref)	ref
II	3.52(3.11,3.98)	<0.001	3.39(2.99,3.85)	<0.001
III	10.12(9.22,11.10)	<0.001	8.01(7.21,8.89)	<0.001
IV	16.14(14.68,17.75)	<0.001	10.86(9.74,12.11)	<0.001
Histology				
Serous	1.00(ref)	ref	1.00(ref)	ref
Mucinous	0.26(0.22,0.29)	<0.001	1.33(1.15,1.54)	<0.001
Endometrioid	0.20(0.18,0.22)	<0.001	0.69(0.61,0.78)	<0.001
Clear	0.49(0.44,0.53)	<0.001	1.49(1.34,1.64)	<0.001
Other epithelial	1.37(1.27,1.48)	<0.001	1.43(1.33,1.55)	<0.001
Non-epithelial	0.27(0.24,0.31)	<0.001	0.86(0.75,0.98)	0.022
Grade				
Well differentiated	1.00(ref)	ref	1.00(ref)	ref
Moderately differentiated	2.95(2.48,3.51)	<0.001	1.99(1.67,2.38)	<0.001
Poorly differentiated/undifferentiated	7.64(6.53,8.95)	<0.001	2.44(2.07,2.88)	<0.001
Unknown	6.86(5.85,8.06)	<0.001	2.29(1.94,2.71)	<0.001
Therapy				
NACT	1.00(ref)	ref	1.00(ref)	ref
Primary surgical therapy	0.49(0.46,0.51)	<0.001	0.89(0.84,0.94)	<0.001
No chemotherapy and/or surgery	0.46(0.43,0.49)	<0.001	1.39(1.30,1.49)	<0.001
Residual				
No residual lesion	1.00(ref)	ref	1.00(ref)	ref
≤1cm	3.37(3.14,3.61)	<0.001	1.65(1.54,1.77)	<0.001
>1cm	3.87(3.63,4.13)	<0.001	1.83(1.71,1.96)	<0.001
Unknown	2.20(2.09,2.31)	<0.001	1.50(1.42,1.58)	<0.001

**Abbreviation NACT**: Neoadjuvant chemotherapy

**Table 4:** Multivariate analyses of the association of treatment delay intervals with OS and CSS

Tumor		Subgroup^c^	OS		CSS	
			HR^a^ (95% CI)	P-value	HR^b^(95% CI)	P-value
Serous	Stage I/II	Intermediate-delay	1.20(0.97,1.47)	0.094	1.09(0.85,1.40)	0.508
		Long-delay	2.65(1.40,5.01)	0.003	2.41(1.13,5.17)	0.023
	Stage III/IV	Intermediate-delay	1.09(1.04,1.15)	<0.001	1.09(1.03,1.15)	0.002
		Long-delay	1.07(0.91,1.25)	0.396	1.09(0.92,1.29)	0.332
Mucinous	Stage I/II	Intermediate-delay	1.28(0.75,2.18)	0.367	1.68(0.84,3.35)	0.144
		Long-delay	2.44(0.86,6.91)	0.092	2.04(0.47,8.87)	0.344
	Stage III/IV	Intermediate-delay	1.01(0.71,1.44)	0.941	0.85(0.55,1.29)	0.444
		Long-delay	0.37(0.13,1.09)	0.073	0.48(0.14,1.66)	0.246
Endometrioid	Stage I/II	Intermediate-delay	1.11(0.74,1.65)	0.618	1.41(0.84,2.37)	0.192
		Long-delay	2.10(0.76,5.77)	0.151	1.63(0.22,11.91)	0.632
	Stage III/IV	Intermediate-delay	1.07(0.80,1.42)	0.664	0.94(0.67,1.32)	0.720
		Long-delay	1.31(0.55,3.13)	0.536	0.80(0.24,2.67)	0.716
Clear	Stage I/II	Intermediate-delay	1.87(1.28,2.73)	0.001	1.91(1.23,2.96)	0.004
		Long-delay	2.44(0.74,8.05)	0.144	2.69(0.63,11.42)	0.180
	Stage III/IV	Intermediate-delay	1.11(0.84,1.45)	0.461	1.28(0.97,1.70)	0.081
		Long-delay	0.49(0.21,1.15)	0.102	0.60(0.25,1.40)	0.236
Other epithelial	Stage I/II	Intermediate-delay	1.40(0.83,2.35)	0.208	0.80(0.38,1.69)	0.558
		Long-delay	1.18(0.16,8.82)	0.872	2.01(0.26,15.59)	0.502
	Stage III/IV	Intermediate-delay	1.04(0.89,1.21)	0.648	1.06(0.90,1.26)	0.488
		Long-delay	0.77(0.47,1.25)	0.290	0.72(0.41,1.27)	0.261
Non-epithelial	Stage I/II	Intermediate-delay	1.01(0.50,2.04)	0.977	0.80(0.31,2.04)	0.636
		Long-delay	1.20(0.28,5.17)	0.802	1.54(0.33,7.11)	0.582
	Stage III/IV	Intermediate-delay	1.31(0.95,1.82)	0.104	1.49(1.03,2.16)	0.032
		Long-delay	0.71(0.35,1.44)	0.342	0.87(0.39,1.93)	0.732

HR**^a^**: compared with immediate-treatment initiation HR**^b^**: compared with immediate-treatment initiation Subgroup**^c^**: immediate: < 1 month, intermediate-delay: 1-2 months, and long-delay: ≥3 months

## Abbreviations

CA 125: cancer antigen 125
CI: confidence interval
COVID-19: coronavirus disease 2019
CSS: cancer-specific survival
HR: hazard ratio
ICD-O-3: International Classification of Oncological Diseases 3
IQR: interquartile range
NACT: neoadjuvant chemotherapy
OS: overall survival
SEER: Surveillance, Epidemiology, and End Results
